# A Neuroimaging Marker Based on Diffusion Tensor Imaging and Cognitive Impairment Due to Cerebral White Matter Lesions

**DOI:** 10.3389/fneur.2019.00081

**Published:** 2019-02-13

**Authors:** Na Wei, Yiming Deng, Li Yao, Weili Jia, Jinfang Wang, Qingli Shi, Hongyan Chen, Yuesong Pan, Hongyi Yan, Yumei Zhang, Yongjun Wang

**Affiliations:** ^1^Department of Neurology, Beijing Tiantan Hospital, Capital Medical University, Beijing, China; ^2^China National Clinical Research Center for Neurological Diseases, Beijing, China; ^3^Center of Stroke, Beijing Institute for Brain Disorders, Beijing, China; ^4^Beijing Key Laboratory of Translational Medicine for Cerebrovascular Disease, Beijing, China; ^5^Department of Interventional Neuroradiology, Beijing Tiantan Hospital, Capital Medical University, Beijing, China; ^6^College of Information Science and Technology, Beijing Normal University, Beijing, China; ^7^Department of Neurology, General Hospital of The Yang Tze River Shipping, Wuhan Brain Hospital, Wuhan, China; ^8^Department of Neuroimaging, Beijing Neurosurgery Institute, Capital Medical University, Beijing, China

**Keywords:** white matter lesions, vascular cognitive impairment, magnetic resonance imaging, diffusion tensor imaging, white matter structural integrity

## Abstract

**Background:** The peak width of skeletonized mean diffusivity (PSMD) is a new, fully automated, robust imaging marker for cerebral small vessel disease (SVD), strongly associated with processing speed. However, it has never been applied to cerebral white matter lesions (WMLs). Our study aimed to investigate the correlation between PSMD and cognition, particularly in the executive function of patients with WMLs.

**Methods:** A total of 111 WML patients and 50 healthy controls (HCs) were enrolled, and their demographic information and cardiovascular disease risk factors were recorded. Subjects were divided into three groups: WMLs with normal cognition (WMLs-NC), WMLs with vascular cognitive impairment (WMLs-VCI), and HCs. They underwent conventional head magnetic resonance imaging and diffusion tensor imaging (DTI), followed by neuropsychological and psychological examinations, including the Montreal Cognitive Assessment (MoCA), and the executive function tests. We compared executive function and PSMD among the three groups and analyzed the correlation between PSMD and cognitive function in all subjects.

**Results:** There were no significant differences in demographic characteristics (age, sex, education level, and cardiovascular disease risk factors) among the three groups (*P* > 0.05), but there were significant differences in global cognition (*P* < 0.0001), executive function (*P* < 0.0001), and PSMD (*P* < 0.0001). The average PSMD value (×10^−4^ mm^2^/s) was 2.40 ± 0.23, 2.68 ± 0.30, and 4.51 ± 0.39 in the HC, WMLs-NC, and WMLs-VCI groups, respectively. There was no correlation between PSMD and cognition in the HC group, but PSMD was significantly correlated with MoCA scores (*r* = −0.3785, *P* < 0.0001) and executive function (*r* = −0.4744, *P* < 0.0001) in the WMLs-NC group and in the WMLs-VCI group (*r* = −0.4448, *P* < 0.0001 and *r* = −0.6279, *P* < 0.0001, respectively).

**Conclusions:** WML patients have higher PSMD and worse cognitive performance than HCs, and PSMD is strongly associated with global cognition and executive functions in WML patients. This result provides new insights into the pathophysiology of cognitive impairment in WML patients. PSMD could be a surrogate marker for disease progression and could thus be used in therapeutic trials involving WML patients.

## Introduction

On computed tomography (CT), cerebral white matter lesions (WMLs) appear as hypodense bilateral and symmetrical areas in the WM of the periventricular region and centrum semiovale (1) and are indicators of cerebral small vessel disease (SVD). The Leukoaraiosis And Disability (LADIS) study confirmed a significant impairment of cognitive function in WML patients (2–4). WMLs are closely correlated to cognitive impairments in attention, executive function, and information processing speed (5). However, some patients may have severe cognitive dysfunction in the absence of widespread WMLs on magnetic resonance imaging (MRI). One of the factors may be the loss of microstructure integrity in the largest part of white matter, which can be visualized with conventional MRI, but can be investigated with DTI.

DTI is a sensitive technique for evaluating disease progression, allowing the quantification of microstructural tissue changes (6). The typical diffusion change pattern in WMLs consists of a decrease in fractional anisotropy (FA) and an increase in mean diffusivity (MD). However, the reliability in multicenter and long-term studies seems questionable. In addition, there are some limitations to the wide application of DTI measures due to the large amount of data postprocessing and the subjective operation errors in methods based on brain regions of interest.

Recently, Baykara et al. (7) proposed the assessment of SVD through DTI parameters which use a WM skeleton to measure the peak width of mean diffusivity in the brain. The new imaging marker was called peak width of skeletonized mean diffusivity (PSMD). Calculations of this marker appear to be robust and promising for studies of large populations. The derived measures were strongly correlated with processing speed and performed better than other neuroimaging markers for SVD, such as WM hyperintensities (WMHs) and the numbers and volumes of lacunes. The method eliminated cerebrospinal fluid contamination and increased the sensitivity in capturing SVD-related changes. A longitudinal analysis revealed the smallest sample size estimate for PSMD when compared with whole brain mean diffusivity peak height, normalized WMH volume, brain parenchymal fraction, processing speed score, or normalized lacune volume. PSMD may thus have a great practical value for clinical research and applications. However, no study has assessed the relationship between PSMD and cognitive function in patients with WMLs, yet.

In the present study, we aimed to examine the relationship between whole WM microstructural integrity, as assessed by PSMD, and cognitive function, in patients with WMLs. We hypothesized that WML patients would show poorer cognitive performance and a higher PSMD than HCs. Further, we aimed to assess the correlation between this new DTI marker and cognition, particularly executive function, in patients with WMLs.

## Materials and Methods

### Subjects

WML patients were recruited from the neurology clinic of the Beijing Tiantan Hospital, Capital Medical University, China, between January 2014 to March 2017. WML was diagnosed independently and unanimously by two radiologists, who visually evaluated the fluid-attenuated inversion recovery (FLAIR) MR images without knowledge of the participants' clinical profiles. The inclusion criteria for WML patients were as follows: (a) age 50–85 years and (b) presence of WMHs on MRI scans, according to a revised version of the Fazekas scale (8). The exclusion criteria were as follows: (a) cardiac or renal failure, cancer, or other severe systemic diseases; (b) unrelated neurological diseases such as epilepsy, traumatic brain injury, or multiple sclerosis; (c) chronic cerebral infarction or other lesions; (d) leukoencephalopathy of non-vascular origin; (e) dementia of non-vascular origin; (f) psychiatric diseases or drug addiction; (g) consciousness disruption or aphasia; or (h) inability or refusal to undergo brain MRI. Initially, 113 WML patients were enrolled. In addition, 48 age-, sex-, and education level-matched normal volunteers were recruited as control subjects. Their age ranged between 50 and 85 years, and their MRI results were normal. The exclusion criteria for the healthy controls were the same as those for the WML patients.

All subjects were administered the Beijing version of the MoCA (9) and the Clinical Dementia Rating (CDR) scale under the supervision of a physician. Based on the results of these cognitive tests, the subjects were divided into three groups: (a) WML patients with normal cognition (WMLs-NC), defined as MoCA ≥ 26 and CDR = 0; (b) WML patients with vascular cognitive impairment (WMLs-VCI), defined as MoCA <26 and CDR > 0; and (c) healthy controls (HC), defined as MoCA ≥ 26 and CDR = 0.

This study was approved by the Ethics Committee of the Beijing Tian Tan Hospital. All patients or their legal representatives provided written informed consent.

### MRI Scanning Protocol

MRI scans of all participants were performed using a 3.0T Signa scanner (Magnetom Trio Tim, Siemens, Germany). The general MRI protocol included the following sequences: T1-weighted 3-dimensional magnetization prepared rapid gradient echo (MPRAGE) sequence (TR/TE/TI 2300/3.28/1200 ms; flip angle 9°; voxel size 1.0 × 1.0 × 1.0 mm), a FLAIR sequence (TR/TE/TI 8000/94/2200 ms; voxel size 1.0 × 1.2 × 5.0 mm, interslice gap 1 mm), and DTI sequences (TR/TE 4900/93 ms; voxel size 2.5 × 2.5 × 2.5 mm; 4 unweighted scans, 30 directions with b-value 1,000 s/mm^2^). Two radiologists blinded to the clinical information assessed the MRI data.

### Processing of PSMD

DTI data were corrected for quick visual inspection to exclude large artifacts. The fully automated calculation of the new marker consists of two steps: Skeletonization of the DTI data and histogram analysis. All study samples were processed with the same pipeline. First, DTI data were skeletonized using the Tract-Based Spatial Statistics procedure included in the Functional Magnetic Resonance Imaging of the Brain (FMRIB) software library (FSL) (10). The fractional anisotropy (FA) data of each subject were projected onto a skeleton derived from a standard space template. The mean diffusivity image was then projected onto the skeleton using FA-derived projection parameters. This process avoids contamination of the skeleton through partial volume effects of cerebrospinal fluid and fornix. The fully automated PSMD calculation pipeline is available at http://www.psmd-marker.com/ as a shell script including all processing steps (including pre-processing). No further human intervention is required during the processing pipeline.

### Neuropsychological Testing

Neuropsychological assessment followed the LADIS protocol (11). In the test battery, the MoCA was considered to be a measure of global cognitive function. Executive function was assessed by computing compound measures from the Stroop color and word test (SCWT), trail-making test (TMT), symbol-digital replacement task (DST), and verbal fluency test (VFT).

### Statistical Analyses

SPSS 23.0 was used for data processing, and SAS 9.4 for statistical analysis. To allow direct comparisons between the imaging marker and neuropsychological tests results, we generated z scores, representing the position of a score value within the score distribution. Executive functions = z scores of ((Stroop3-2) + (TMB-TMA) + symbol digit + verbal fluency)/4.The sign of each z-score was changed if necessary, to make positive scores correspond to better performance. The results of the MoCA were also transformed into z scores.

The numerical variables were reported as mean ± standard deviation (SD), and as median and interquartile ranges for parameters with skewed distributions. Normally distributed continuous variables were compared by one-way analysis of variance(ANOVA), and the Kruskal-Wallis test was used to compare non-normally distributed variables. A chi squared test was used to compare categorical variables. Multivariate regression analysis was used to assess the relative contribution of PSMD measures to performance in different cognitive domains. Regression analysis was performed with two levels of adjustment for covariates: Model 1 adjusted for age, sex, and level of education; model 2 extended model 1 by the addition of hypertension, diabetes, hyperlipidemia, coronary heart disease, smoking status, drinking, and BMI. Furthermore, using Pearson correlation analysis, we examined the association between PSMD value and cognitive functions. *P*-values <0.05 were considered statistically significant.

## Results

A total of 163 subjects were enrolled in this study. Among them, there were 35 and 78 patients in the WMLs-NC and WMLs-VCI groups, respectively, and 48 healthy controls. The baseline characteristics of all subjects are listed in Table 1. There were no statistically significant differences in age, sex, years of education, or incidence of cerebral vessel risk factors in terms of hypertension, hyperlipidemia, coronary heart disease, smoking, drinking, and BMI among the three groups (*P* < 0.05).

**Table 1:** Characteristics of the study population.

**Characteristics**	**HC (*n* = 48)**	**WMLs-CN (*n* = 35)**	**WMLs-VCI (*n* = 78)**	***P*-value**
**DEMOGRAPHICS**				
Male	24 (50.00)	18 (51.43)	42 (52.50)	0.9631^a^
Age, y	56.83 ± 4.72	61.94 ± 8.50	63.03 ± 9.37	0.0031^b^
Years of education	12.38 ± 3.19	11.54 ± 3.06	11.48 ± 3.05	0.3175 ^b^
**VASCULAR RISK FACTORS**				
Hypertension	21 (43.75)	18 (51.43)	47 (58.97)	0.0902^a^
Diabetes	8 (16.67)	7 (20.00)	18 (23.08)	0.3337^a^
Hyperlipidemia	16 (33.33)	12 (34.29)	30 (38.46)	0.1270^a^
Coronary heart disease	5 (10.42)	5 (14.29)	14 (17.95)	0.2300^a^
Smoking	19 (39.58)	10 (28.57)	19 (24.36)	0.2189^a^
Drinking	13 (27.08)	8 (22.86)	20 (25.64)	0.9498^a^
BMI	24.29 ± 2.24	24.25 ± 2.08	24.77 ± 2.33	0.3125^b^

Categorical variables are expressed as number (percentage), and continuous ones as mean ± SD. HC, healthy controls; WMLs-CN, cognitively normal white matter lesion patients; WMLs-VCI, white matter lesion patients with vascular cognitive impairment; BMI, body mass index.

a The P-value was obtained by chi squared (χ^2^) test.

b *The P-value was obtained by ANOVA*.

As shown in Table 2, there were significant differences in cognitive status among the three groups, as measured by the MoCA z score (*P* < 0.0001), and in executive functions (*P* < 0.0001). There were significant differences in PSMD among the three groups (*P* < 0.0001). The mean PSMD values were 2.40 ± 0.23 × 10^−4^ mm^2^/s, 2.68 ± 0.30 × 10^−4^ mm^2^/s, and 4.51 ± 0.39 × 10^−4^ mm^2^/s in the healthy controls, WMLs-NC, and WMLs-VCI, respectively.

**Table 2:** Cognitive function measures and the peak width of skeletonized mean diffusivity in the study population.

**Characteristics**	**HC (*n* = 48)**	**WMLs-CN (*n* = 35)**	**WMLs-VCI (*n* = 78)**	***P*-value**
**GLOBAL COGNITIVE FUNCTION**				
MoCA	27.81 ± 1.53	26.74 ± 1.09	20.76 ± 3.46	<0.0001
z score	0.00 ± 2.43	0.00 ± 1.00	0.01 ± 1.01	<0.0001
**EXECUTIVE FUNCTIONS**				
SCWT- B (s)	43.60 ± 1.01	44.37 ± 2.50	68.04 ± 7.58	<0.0001
SCWT- C (s)	63.30 ± 4.20	64.15 ± 2.72	93.13 ± 3.40	<0.0001
SCWT (C-B) (s)	19.40 ± 4.14	19.78 ± 3.07	25.09 ± 4.39	<0.0001
TMT-A (s)	30.77 ± 0.87	32.05 ± 1.77	42.54 ± 1.92	<0.0001
TMT-B (s)	80.49 ± 0.74	81.17 ± 1.47	89.86 ± 5.90	<0.0001
TMT (B–A) (s)	49.72 ± 0.96	49.12 ± 1.22	47.32 ± 4.27	0.0347
DST	47.90 ± 2.98	45.51 ± 2.29	33.94 ± 4.92	<0.0001
VFT	11.07 ± 0.84	10.83 ± 0.98	7.81 ± 1.28	<0.0001
z scores	0.00 ± 2.04	−0.53 ± 3.42	−0.66 ± 1.57	<0.0001
PSMD (10^−4^mm^2^/s)	2.40 ± 0.23	2.68 ± 0.30	4.51 ± 0.39	<0.0001

*Variables and z scores are shown as mean ± SD. HC, healthy controls; WMLs-CN, cognitively normal white matter lesion patients; WMLs-VCI, white matter lesion patients with vascular cognitive impairment; MoCA, Montreal Cognitive Assessment; SCWT, Stroop color and word test; TMT, trail-making test; DST, symbol-digital replacement task, VFT, verbal fluency test; PSMD, peak width of skeletonized mean diffusivity*.

As seen in Table 3, when assessing the relationship between PSMD and global cognitive function, no significant associations were seen with test results in the HC or WMLs-NC groups after correction for age, sex, and education level (model 1) or after adjustment for hypertension, diabetes, hyperlipidemia, coronary heart disease, smoking status, drinking, and BMI (model 2). However, the association with global cognitive function in the WMLs-VCI group was significant in the fully adjusted model (ß = -0.513; standard error [SE] = 0.091; *P* < 0.001). When assessing the relationship between PSMD and executive function, the association remained significant in both model 1 and model 2. In the latter model the coefficients were: HCs: ß = −2.155; SE = 0.714; *P* = 0.005; WMLs-NC: ß = −1.629; SE = 0.741; *P* = 0.039; and WMLs-VCI: ß = −0.372; SE = 0.107; *P* < 0.001.

**Table 3:** Associations between the peak width of skeletonized mean diffusivity and cognitive performance.

**Cognitive function**	**HC**	**WMLs-CN**	**WMLs-VCI**
**GLOBAL COGNITIVE FUNCTION (Z SCORES)**			
**Model 1**			
ß	−0.598	−1.505	−0.558
SE	1.484	0.953	0.088
*P*	0.689	0.125	< 0.001
**Model 2**			
ß	−0.272	−1.549	−0.513
SE	1.690	1.149	0.091
*P*	0.873	0.191	< 0.001
**EXECUTIVE FUNCTIONS (Z SCORES)**			
**Model 1**			
ß	−1.838	−1.583	−0.382
SE	0.657	0.555	0.100
*P*	0.008	0.008	< 0.001
**Model 2**			
ß	−2.155	−1.629	−0.372
SE	0.714	0.741	0.107
*P*	0.005	0.039	0.001

*Model 1: adjusted for age, sex, and level of education. Model 2: same as model 1, additionally adjusted for hypertension, diabetes, hyperlipidemia, coronary heart disease, smoking status, drinking and BMI. SE, standard error*.

As seen in Table 4, there were no significant associations between PSMD and cognitive performance in the healthy control samples (*P* = 0.56–0.88). Negative correlation was found between PSMD and global and executive function in the WMLs-CN samples, with correlation coefficients −0.3785 and −0.4744, respectively, and also in the WMLs-VCI samples, with correlation coefficients −0.4448 and −0.6279, respectively.

**Table 4:** Correlation between cognitive function and the peak width of skeletonized mean diffusivity.

**Cognitive function**	**HC**	**WMLs-CN**	**WMLs-VCI**
**GLOBAL COGNITIVE FUNCTION (Z SCORES)**			
*r*	0.0355	−0.3785	−0.4448
*P*	0.88	<0.0001	<0.0001
**EXECUTIVE FUNCTIONS (Z SCORES)**			
*r*	−0.0979	−0.4744	−0.6279
*P*	0.56	<0.0001	<0.0001

*HC, health controls; WMLs-CN, cognitively normal white matter lesion patients; WMLs-VCI, white matter lesion patients with vascular cognitive impairment. r, Pearson's correlation coefficient*.

**Figure 1:**
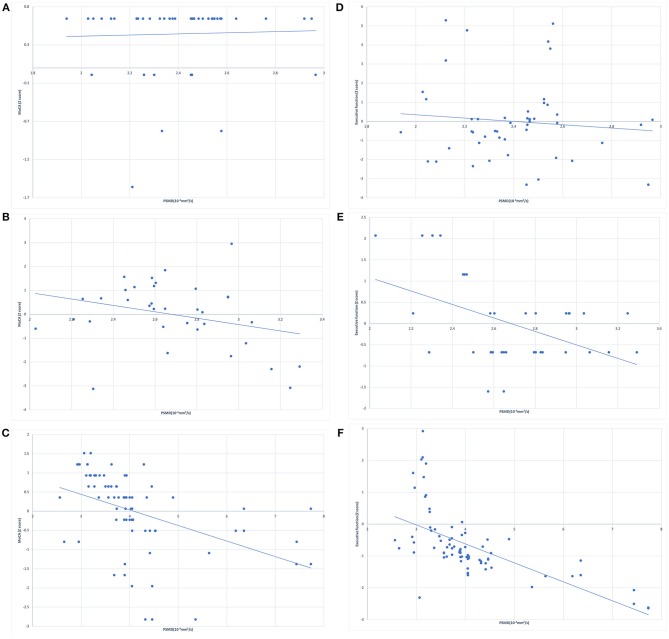
Simple linear regression between peak width of skeletonized mean diffusivity (PSMD) and z scores of MoCA in **(A)** healthy controls, **(B)** cognitively normal WML patients, **(C)** WML patients with vascular cognitive impairment, and for the z scores of executive function in **(D)** healthy controls, **(E)** cognitively normal WML patients, **(F)** the WML patients with vascular cognitive impairment.

The correlation analysis between cognitive performance and PSMD in healthy controls and WML patients is shown in Figure 1. Linear regression showed no significant association between PSMD and MoCA scores (Figure 1A) or executive function (Figure 1B) in the healthy controls. The WMLs-CN group showed associations between PSMD and MoCA scores (Figure 1C) as well as executive function (Figure 1D). The WMLs-VCI group showed strong associations between PSMD and MoCA scores (Figure 1E) as well as executive function (Figure 1F).

## Discussion

In this study, we analyzed PSMD and cognitive function in HC subjects and WMLs patients, with or without cognitive impairment. We also investigated the relationship between PSMD and cognitive functions.

There were no significant differences in age, sex, years of education, and vascular risk factors between the WML patients and the healthy controls, largely eliminating the influence of possible confounders on cognitive assessment. Extensive neuropsychological assessment was performed by two investigators, and multivariate regression analysis included adjustment for potential confounders. Global cognitive function was measured using MoCA scores, which have been proposed as a screening tool for vascular cognitive impairment (12). Pasi et al. demonstrated that DTI-measured WM microstructural damage is more related to MoCA results than to mini mental state examination performances in SVD patients (13), indicating that MoCA is suitable for the cognitive screening of patients with small vessel disease. Yuan et al. demonstrated that the cognitive domains affected in patients with WMLs were attention, executive function, and information processing speed (2012). The loss of memory is not common in patients with cognitive impairment due to WMLs, which is one of the differences between vascular dementia and Alzheimer's disease (14).

In our study, executive function was assessed by computing compound measures from the SCWT, TMT, DST, and VFT, which examine the cognitive domain of psychomotor speed, fluency, concept shifting, and attention. We found that the global cognitive and executive functions of WML patients were significantly worse than those of healthy subjects. Further, WMLs-NC patients had better executive function performance than WMLs-VCI patients. These findings are consistent with those of other studies (15).

The characteristics of cognitive impairment in individuals with WMLs depend on the location, degree, and size of the lesions. Diffusion tensor tractography study showed that the location of WMLs was related to the damaged cognitive domain (2). As cognitive disturbances in subjects with cerebral small vessel disease are related to microstructural integrity of multiple WM fibers (within WMH and normal-appearing WM) connecting different cortical and subcortical regions, we examined the relation between the microstructural integrity of the whole WM and cognitive performance in subjects with WMLs. In addition, whole brain histogram analysis is particularly appropriate when quantifying total disease burden (16). In this study, we measured PSMD as an imaging marker to measure the microstructural integrity of the whole WM in HC subjects and WML patients. Our results showed that WML patients had higher PSMD values than control subjects. Moreover, we found that in WML patients the severity of cognitive impairment increased with PSMD.

DTI measures are more sensitive than conventional MRI markers in capturing changes associated with SVD. Histograms of MR parameter values measured in the whole brain are increasingly being used to characterize subtle disease that affects large parts of the brain. Studies have shown that histogram peak height measures were associated with cognitive function and can capture disease burden in SVD (17, 18). PSMD is a combination of DTI, skeletonization and histogram analysis of WM tracks, and is therefore superior in assessing the burden of disease (19).

In this study, we examined the relationship between PSMD and cognitive performance in the three groups. We found that there were no significant associations between PSMD and cognition performance in the healthy controls. However, in the WML group, PSMD was associated with executive dysfunction, a pattern that has been associated with SVD (20). The associations were also reflected in the MoCA scores, which give a global measure of cognitive function. Our study found a clear inverse relationship between PSMD and cognition: High PSMD values were associated with lower scores for cognitive functions, and especially for executive performance. As PSMD is a whole-brain measure computed from DTI scans, but not a local estimate of possible changes in the microstructure within the brain, subjects with the same WMH loads may show different cognitive performances. We demonstrated that PSMD was highly correlated with executive function in WML patients. Moreover, the correlation was more significant in WML patients with cognitive impairment. This imaging marker is therefore highly sensitive to vascular cognitive impairment and could therefore be used in addition to conventional MRI to investigate cognitive dysfunction.

A major strength of this study is that all subjects were assessed by multiple MRI sequences, including 3D T1, FLAIR, and 30-direction DTI acquisition. Therefore, accurate and comprehensive original image data were acquired. Another strength is the use of novel imaging techniques: Image data are processed by the online scanning software. All the processing steps are simple and fully automated, without any manual intervention. Furthermore, our study is a single-center study, with all subjects examined by only two investigators, using manual segmentation of the WML, without prior knowledge of the clinical data.

Our study also had limitations. First, the study was hospital-based, and patients who did not meet the inclusion criteria were excluded. This may result in selection bias and influence the measurement of cognitive function in WML patients. Second, PSMD mostly reflects SVD-related alterations or primary neurodegenerative pathology, and the subjects should be grouped based on the degree of WM damage, but this could not be done in the present study. In future studies, it is desirable to combine the measurements of cognitive impairment and WMH load with PSMD, in order to elucidate the correlations between brain microstructural integrity and cognitive function associated with WMLs. Third, our study is cross-sectional, and the causal interpretation of the results is limited. Future studies with larger sample sizes may further clarify the interactions between cognitive impairment and brain microstructural characteristics.

To summarize, the present study comprehensively investigated the characteristics of PSMD in WML patients and compared them with those of HC subjects. We demonstrated that PSMD is significantly correlated with cognitive impairment in WML patients. Our findings suggest that microstructural integrity of the whole WM should be considered when investigating the relationship between WMLs and cognitive function. PSMD could therefore serve as an addition to a conventional MRI in order to investigate cognitive dysfunction. This result provides new insights into the pathophysiology of cognitive impairment in WML patients. PSMD could be a surrogate marker for disease progression and could thus be used in therapeutic trials involving WML patients.

## Author Contributions

NW and YZ designed the study and drafted the manuscript. YD, WJ, QS, and JW performed the measurements and collected the data. HC performed the brain MRI data acquisition. LY participated in the brain imaging data analysis. YP and HY performed the statistical analysis. YZ and YW participated in the critical discussion of the manuscript.

### Conflict of Interest Statement

The authors declare that the research was conducted in the absence of any commercial or financial relationships that could be construed as a potential conflict of interest.
